# Kisspeptin modulates sexual and emotional brain processing in humans

**DOI:** 10.1172/JCI89519

**Published:** 2017-01-23

**Authors:** Alexander N. Comninos, Matthew B. Wall, Lysia Demetriou, Amar J. Shah, Sophie A. Clarke, Shakunthala Narayanaswamy, Alexander Nesbitt, Chioma Izzi-Engbeaya, Julia K. Prague, Ali Abbara, Risheka Ratnasabapathy, Victoria Salem, Gurjinder M. Nijher, Channa N. Jayasena, Mark Tanner, Paul Bassett, Amrish Mehta, Eugenii A. Rabiner, Christoph Hönigsperger, Meire Ribeiro Silva, Ole Kristian Brandtzaeg, Elsa Lundanes, Steven Ray Wilson, Rachel C. Brown, Sarah A. Thomas, Stephen R. Bloom, Waljit S. Dhillo

**Affiliations:** 1 Investigative Medicine,; 2 Division of Brain Sciences,and; 3 Imanova Centre for Imaging Sciences, Imperial College London, London, United Kingdom.; 4 Statsconsultancy Ltd., Amersham, Bucks, United Kingdom.; 5 Department of Neuroradiology, Imperial College Healthcare NHS Trust, London, United Kingdom.; 6 Centre for Neuroimaging Sciences, King’s College London, London, United Kingdom.; 7 Department of Chemistry, University of Oslo, Oslo, Norway.; 8 Institute of Chemistry, University of Sao Paulo, Sao Carlos, Brazil.; 9 King’s College London, Faculty of Life Sciences & Medicine, Institute of Pharmaceutical Science and Department of Physiology, London, United Kingdom.

## Abstract

**BACKGROUND.** Sex, emotion, and reproduction are fundamental and tightly entwined aspects of human behavior. At a population level in humans, both the desire for sexual stimulation and the desire to bond with a partner are important precursors to reproduction. However, the relationships between these processes are incompletely understood. The limbic brain system has key roles in sexual and emotional behaviors, and is a likely candidate system for the integration of behavior with the hormonal reproductive axis. We investigated the effects of kisspeptin, a recently identified key reproductive hormone, on limbic brain activity and behavior.

**METHODS.** Using a combination of functional neuroimaging and hormonal and psychometric analyses, we compared the effects of kisspeptin versus vehicle administration in 29 healthy heterosexual young men.

**RESULTS.** We demonstrated that kisspeptin administration enhanced limbic brain activity specifically in response to sexual and couple-bonding stimuli. Furthermore, kisspeptin’s enhancement of limbic brain structures correlated with psychometric measures of reward, drive, mood, and sexual aversion, providing functional significance. In addition, kisspeptin administration attenuated negative mood.

**CONCLUSIONS.** Collectively, our data provide evidence of an undescribed role for kisspeptin in integrating sexual and emotional brain processing with reproduction in humans. These results have important implications for our understanding of reproductive biology and are highly relevant to the current pharmacological development of kisspeptin as a potential therapeutic agent for patients with common disorders of reproductive function.

**FUNDING.** National Institute for Health Research (NIHR), Wellcome Trust (Ref 080268), and the Medical Research Council (MRC).

## Introduction

Unraveling the intrinsic links among sex, emotion, and reproduction relies on a focused exploration of putative factors. Identifying a factor that unites these fundamental components of human behavior has until now remained elusive. The reproductive hormone kisspeptin (encoded by *KISS1*) has recently emerged as a crucial activator of the reproductive axis acting in the hypothalamus to stimulate downstream secretion of reproductive hormones (1–3). However, the expression of *KISS1* and its cognate receptor (encoded by *KISS1R*) is not limited to the hypothalamus. Significant *KISS1/KISS1R* expression has been reported in limbic brain structures in rodents (4–8) and humans (9, 10), but little is known about the role of kisspeptin in these areas. The limbic system has established roles in emotional and reproductive behavior and so may provide a physiological framework uniting sex, emotion, and reproduction in humans. In this study, we employed kisspeptin administration to healthy men to explore this further. We hypothesized that kisspeptin administration modulates limbic brain activity in response to sexual and emotional stimuli and associates with related behavioral measures.

**Figure 1:**
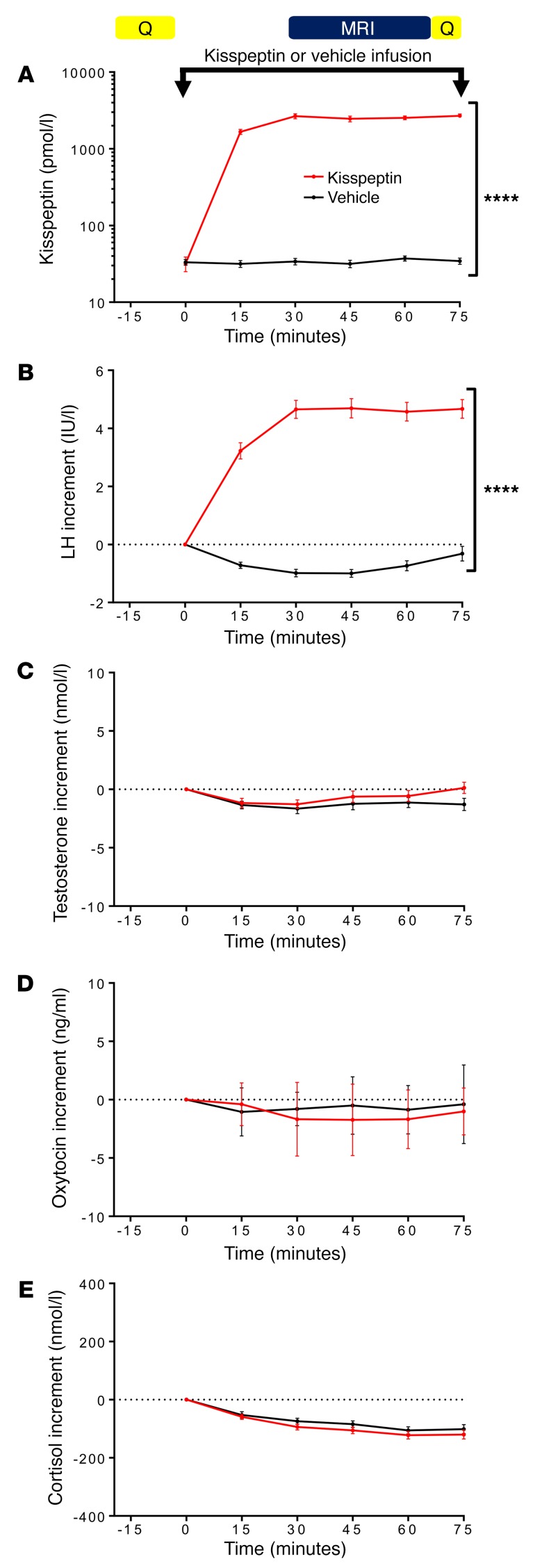
Experimental protocol and effects of kisspeptin administration on hormone levels. Twenty-nine healthy young men participated in a randomized, double-blinded, 2-way crossover, placebo-controlled study. They participated in 2 study visits: one for intravenous administration of kisspeptin (1 nmol/kg/h) and one for intravenous administration of an equivalent volume of vehicle for 75 minutes. Participants completed baseline and intrainfusion questionnaires (Q) and underwent functional MRI scanning while performing image tasks (see Methods). (**A**) Kisspeptin infusion resulted in increased circulating kisspeptin levels reaching a plateau at 30 minutes after initiation. Therefore, there were stable circulating kisspeptin levels during the fMRI and intrainfusion psychometric assessments (*n* = 29). (**B**) In parallel, kisspeptin increased circulating LH levels (*n* = 29). (**C**–**E**) Kisspeptin had no effect on circulating testosterone (*n* = 29), oxytocin (*n* = 13), or cortisol levels (*n* = 29). Data depict mean ± SEM. ****P < 0.0001, 2-way ANOVA.

To test our hypothesis, we performed a randomized, double-blinded, 2-way crossover, placebo-controlled study in 29 healthy heterosexual young men to explore the effects of kisspeptin administration on limbic brain activity in response to sexual and emotional stimuli as well as additional psychometric measures (protocol summarized in Figure 1 and participant characteristics in Supplemental Tables 1 and 2; supplemental material available online with this article; https://doi.org/10.1172/JCI89519DS1). We administered kisspeptin or vehicle, used emotional images to trigger underlying limbic brain activity, and mapped kisspeptin’s modulation of this activity using functional MRI (fMRI). During the emotional images task, participants viewed sexual-, nonsexual couple-bonding–, negative-, and neutral-themed images. Following this, images of happy, fearful, and neutral emotional faces were presented to participants.

## Results

### Kisspeptin administration increased circulating kisspeptin, but not testosterone, oxytocin, or cortisol.

Baseline kisspeptin, gonadotrophin, and testosterone levels were equivalent between study visits in the 29 healthy heterosexual young men (Supplemental Table 2). Kisspeptin administration led to significant increases in circulating kisspeptin levels, reaching steady-state levels for the duration of the fMRI and psychometric questionnaire sessions, as expected (Figure 1A). Importantly, the fMRI questionnaire sessions were performed before any downstream increases in testosterone (Figure 1C), which are known to occur after 90 minutes following kisspeptin exposure in humans (11, 12). Kisspeptin administration had no effect on other relevant hormones that could affect limbic activity, including oxytocin and cortisol (Figure 1, D and E).

### Kisspeptin administration enhanced limbic brain activity in response to sexual images, which correlated with psychometric measures.

**Figure 2:**
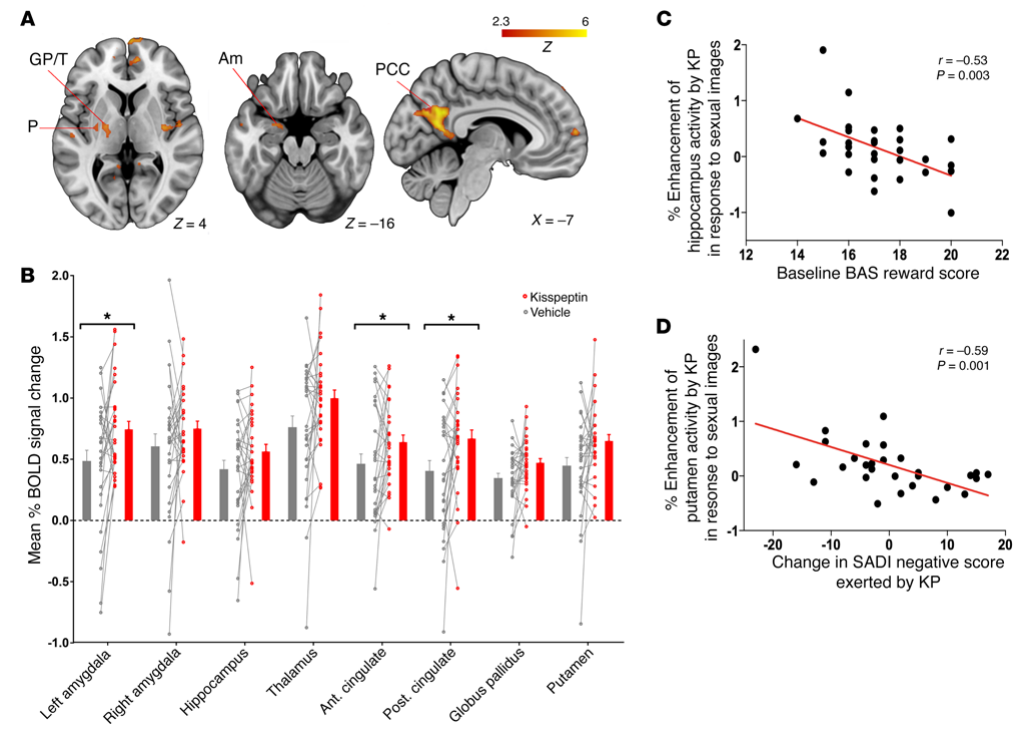
Sexual images, effects of kisspeptin on brain activity, and correlations with reward and sexual aversion. (**A**) Whole-brain analysis of enhanced activity by kisspeptin administration in response to sexual images. Am, amygdala; GP, globus pallidus; PCC, posterior cingulate cortex; P, putamen; T, thalamus. (**B**) Percentage of mean BOLD signal change in a priori limbic and paralimbic anatomically defined ROIs in response to sexual images. Data depict within-participant paired raw data, mean ± SEM. *P < 0.05, paired 2-sided *t* test. Ant., anterior, Post., posterior. (**C**) Correlation between baseline reward score (BAS reward score) and enhancement of hippocampal activity by kisspeptin in response to sexual images. (**D**) Correlation between change in sexual aversion (SADI-negative) and putamen enhancement by kisspeptin (KP) in response to sexual images. Partial correlation testing, adjusted for visit order. *n* = 29.

Heterosexual young men viewing sexual images exhibited enhanced activity in key limbic and paralimbic structures during kisspeptin compared with vehicle administration (Figure 2A and Supplemental Table 3). In keeping with this, analysis of a priori limbic and paralimbic anatomically-defined regions of interest (ROIs) (Supplemental Figure 1) revealed that, in response to sexual images, kisspeptin enhanced brain activity in the anterior and posterior cingulate as well as the left amygdala (Figure 2B), regions expressing kisspeptin and kisspeptin receptors (4–10) and consistent with areas of activation by sexual stimuli in previous physiological studies (13–15).

Next, we correlated brain activity in the anatomical ROIs (Supplemental Figure 1) with psychometric measures to explore functional relevance (while correcting for multiple comparisons; see Methods), as the limbic system has established roles in reward- and drive-oriented behaviors (16). In response to sexual images, kisspeptin activated key limbic structures more in participants with lower baseline reward-behavior scores (Behavioral Activation System [BAS] reward vs. hippocampus, *r* = –0.53, *P* = 0.003 (Figure 2C); vs. right amygdala, *r* = –0.49, *P* = 0.008) and lower baseline drive-behavior scores (BAS drive vs. hippocampus, *r* = –0.47, *P* = 0.009; vs. posterior cingulate, *r* = –0.52, *P* = 0.004).

In addition, the more kisspeptin enhanced activity in response to sexual images (in several limbic structures typically activated by sexual arousal in previous studies; refs. 13–15), the less aversion to sex participants exhibited (Sexual Arousal and Desire Inventory [SADI] negative vs. putamen, *r* = –0.59, *P* = 0.001, (Figure 2D); vs. anterior cingulate, *r* = –0.56, *P* = 0.008; vs. posterior cingulate, *r* = –0.53, *P* = 0.003; vs. globus pallidus, *r* = –0.47, *P* = 0.01). Kisspeptin had no effect on other psychometric measures of sexual arousal, or attention and anxiety which could have confounded brain activity results (Supplemental Figure 2).

### Kisspeptin administration enhanced limbic brain activity in response to nonsexual couple-bonding images, which correlated with psychometric measures.

**Figure 3:**
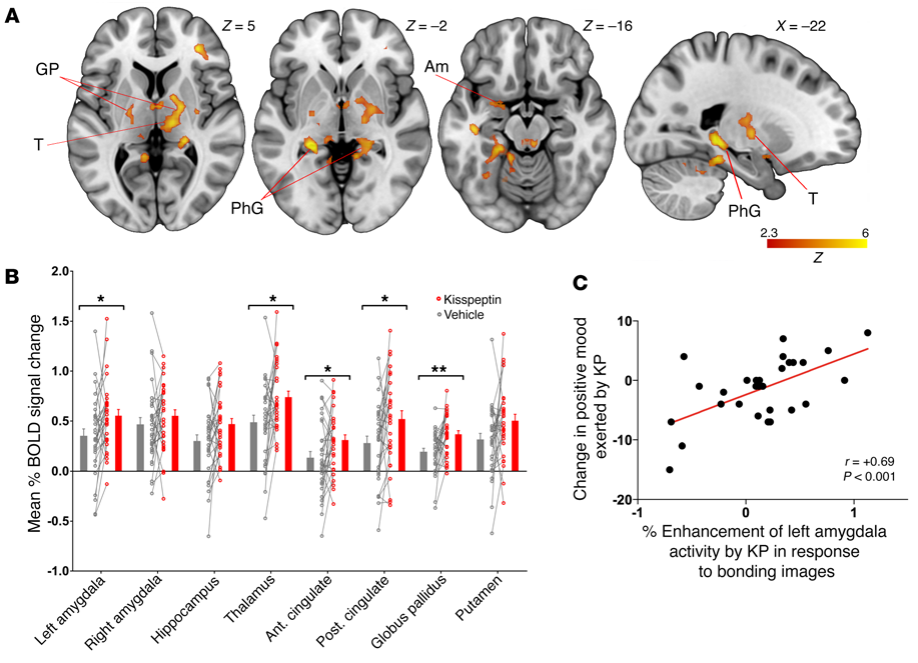
Nonsexual couple-bonding images, effects of kisspeptin on brain activity, and correlation with mood. (**A**) Whole-brain analysis of enhanced activity by kisspeptin administration in response to nonsexual couple-bonding images. PhG, parahippocampal gyrus. (**B**) Mean percentage of BOLD signal change in a priori limbic and paralimbic anatomically defined ROIs in response to nonsexual couple-bonding images. Data depict within-participant paired raw data, mean ± SEM. *P < 0.05; **P < 0.01, paired 2-sided *t* test. (**C**) Correlation between enhancement of amygdala activity by kisspeptin in response to couple-bonding images and change in positive mood exerted by kisspeptin. Partial correlation testing, adjusted for visit order. *n* = 29.

On viewing nonsexual couple-bonding–themed images, kisspeptin administration resulted in brain activation patterns similar to those observed above in response to sexual images, including activation of the anterior and posterior cingulate and amygdala (Figure 3, A and B). However, kisspeptin also markedly enhanced activity in the thalamus and globus pallidus: regions previously implicated in “romantic love” (17, 18) and expressing kisspeptin receptors in humans (9) (Figure 3B). Likewise, the amygdala is implicated in bonding (18), and we observed that kisspeptin’s enhanced activation of the amygdala in response to bonding images related to improvements in positive mood (*r* = 0.69, *P* < 0.001, Figure 3C).

### Kisspeptin administration did not modulate limbic brain activity in response to other themed images or during a battery of nonlimbic tasks.

Kisspeptin administration did not modulate limbic brain activity in response to negative-, neutral-, happy-, or fearful-themed images (Supplemental Figures 3 and 4 and Supplemental Table 3). In addition, kisspeptin had no effect on brain activity during a battery of nonlimbic tasks (visual, auditory, motor, language, calculation; Supplemental Figure 5).

### Kisspeptin administration enhanced frontal brain activity in response to negative images and reduced negative mood.

**Figure 4:**
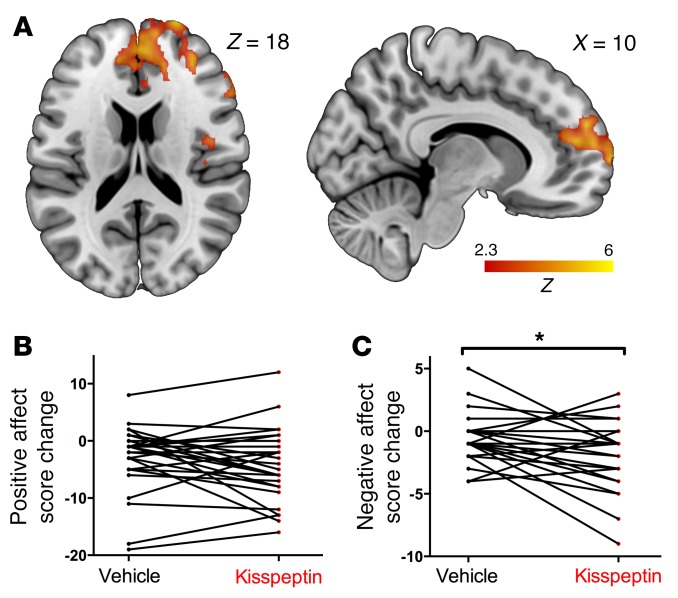
Negative images, effects of kisspeptin on brain activity, and mood. (**A**) Whole-brain analysis of enhanced activity by kisspeptin administration in response to negative images. (**B**) Effect of vehicle and kisspeptin on positive affect score, assessed using the PANAS, presented as score change from baseline for each participant. (**C**) Effect of vehicle and kisspeptin on negative affect score (PANAS), presented as score change from baseline for each participant. *P < 0.05, multi-level linear regression, adjusted for visit order. *n* = 29.

Although kisspeptin had no effect on limbic structures when viewing negative images in our study, kisspeptin instead enhanced activity in a region around the frontal pole extending caudally to the paracingulate gyrus (Figure 4A and Supplemental Table 3), involving structures important in human negative-mood regulation (19) and expressing kisspeptin receptors (9). In keeping with this, we observed that although kisspeptin administration did not affect positive mood (Figure 4B), kisspeptin administration elicited a reduction in negative mood (*P* = 0.031, Figure 4C).

## Discussion

In this study, we demonstrate that the reproductive hormone kisspeptin enhances limbic brain activity specifically in response to sexual and bonding stimuli and that these responses correlate with psychometric measures of sexual and emotional processing. Sexual and emotional responses are fundamental drivers of human behavior, and the links among sex, bonding, and reproduction ultimately ensure the survival of most mammalian species (20). However, the pathways involved are multiple, complex, relatively poorly understood, and involve reproductive and metabolic hormones, pheromones, neuronal networks, peripheral organs, and various sensory signals, among others. Our data suggest a potential role for kisspeptin as an important neuromodulator, linking sexual and emotional brain processing with the reproductive axis.

Visually evoked sexual arousal is a frequent occurrence in men, and brain activity associated with visual sexual stimuli have been explored in several previous studies. These studies have examined a wide range of brain structures in response to sexual-themed images and revealed a processing network involving structures including the hypothalamus, amygdala, thalamus, cingulate, insula, precentral gyrus, and occipital cortex (13–15, 21–28). Furthermore, activations in structures including the thalamus and cingulate correlate with physiological sexual arousal (as assessed by penile tumescence) (13). The involvement of these structures therefore suggests cognitive (cingulate, thalamus), emotional (amygdala, insula), motivational (precentral gyrus), and physiological (thalamus) components to sexual arousal from the appraisal of a stimulus as sexual through to the autonomic activation in readiness for sexual behavior (13, 27, 28).

Kisspeptin sits at the apex of the reproductive axis, above gonadal hormones such as testosterone that are known to be involved in sexual and emotional processing (29). Kisspeptin signaling is also essential in the “timing” of reproduction, from regulating gonadotropin-releasing hormone (GnRH) pulsatility, oestrous cyclicity, and sexual development to aging (30). In our study, kisspeptin enhanced activity in key limbic and paralimbic structures when heterosexual young men viewed sexual images. These included the anterior and posterior cingulate as well as the left amygdala, consistent with areas of activation observed in the above studies (13–15, 21–28) and with regions expressing kisspeptin and kisspeptin receptors (4–10). Therefore, we demonstrate that kisspeptin administration enhances activation in key established areas of the sexual-processing network.

It is interesting that, although kisspeptin enhanced activity in both the right and left amygdala, this only reached statistical significance on the left. Although the right amygdala often shows greater enhancement during image-related emotion stimulation (31, 32), the left amygdala is more often engaged in sexual (14) and emotional processing in men (33), and so in this study, kisspeptin may be preferentially acting on the left amygdala in keeping with these studies. Future studies may seek to examine whether there is a lateralization of kisspeptin and kisspeptin receptor expression in the amygdala to address this further.

We then proceeded to correlate modulations in brain activity with our psychometric data to provide functional relevance. Interestingly, kisspeptin’s enhancement of several structures of the sexual-processing network (including the cingulate, putamen, and globus pallidus) correlated with reduced sexual aversion, suggesting a role for kisspeptin in sexual disinhibition.

Drive and reward traits are primary components of BAS, which has key functions in bringing the individual together with biological rewards such as sex and food (34, 35). Furthermore, previous studies have shown that these traits predict fMRI responses to appetizing foods (36) and sexual images (37). The neural substrate of the BAS comprises structures belonging to the mesolimbic reward and fronto-striatal-amygdala-midbrain networks (36, 37). Intriguingly, in our study, kisspeptin activated key components related to these networks (including the hippocampus, amygdala, and cingulate) more in participants with lower baseline drive and reward traits in response to viewing sexual images. It is interesting to speculate as to a functional reason for this. Kisspeptin was able to enhance activity in components of this reward circuitry more in participants who were less reward responsive. This could serve as a functional mechanism for enhancing reward-system activity during sexual arousal (in those generally less responsive to reward), so as to drive a desire for reproduction in these individuals.

Collectively, these data suggest that kisspeptin not only enhances activation in established structures of sexual arousal, but that this activation correlates with behavioral measures of reward, drive, and sexual aversion. Consistent with the expression pattern of kisspeptin and its cognate receptor in these regions (4–10), we provide evidence for kisspeptin as a neuroendocrine modulator of the human brain sexual-processing network.

In addition to sexual stimulation, an important precursor to reproduction is the desire to bond with a partner. Studies of bonding have examined different types: romantic love, maternal love, and unconditional love. Studies of romantic love demonstrate activations in dopamine-rich and basal ganglia structures such as the putamen, thalamus, and globus pallidus (17, 38, 39), which are associated with reward (40), pair-bonding (41), and euphoria (16). In addition, activations are commonly seen in areas associated with mental associations (e.g., hippocampus and thalamus) and emotional areas also implicated in sexual processing (e.g., cingulate and amygdala) (17, 38, 39, 42). There is substantial overlap with the processing networks in maternal love, including the cingulate, globus pallidus, amygdala, and dopaminergic brain areas (43). Activations are also observed in reward and dopamine-rich areas (e.g., globus pallidus and cingulate) in unconditional love (44). Taken together, these studies suggest a common subcortical dopaminergic reward-related brain system as well as higher-order cortical cognitive centers driving love and bonding.

In the current study, kisspeptin modulated the response to bonding images in regions similar to those seen with sexual images, including the anterior and posterior cingulate and amygdala, with the addition of activation in the thalamus and globus pallidus. These activations by kisspeptin match regions implicated in romantic love, maternal love, and even unconditional love in the aforementioned studies as well as being sites of kisspeptin and kisspeptin receptor expression (4–10). Furthermore, we observed that kisspeptin’s enhanced activation of the amygdala in response to bonding images correlated with improvements in positive mood. Taken together, these data demonstrate that kisspeptin enhanced activity in key “romance and bonding” structures in response to viewing couple-bonding images and that this correlated with improved positive mood. We therefore provide evidence in humans of a role for kisspeptin in the processing of sexual and bonding stimuli, both of which are critical in driving reproduction at a behavioral level.

Consistent with the correlation between kisspeptin’s enhancement of amygdala activity and improvements in positive mood in humans above, recent rodent data suggest antidepressant-like effects for kisspeptin via the serotonergic system (45). In our study, kisspeptin enhanced prefrontal activity in response to negative images; a region expressing kisspeptin receptors (9). This is consistent with studies of negative-evoked stimuli, demonstrating predominant activation in prefrontal regions commonly implicated in response inhibition and self-control. Greater activity in these regions assists internalized representations of safety to minimize fear and anxiety to negative stimuli (19). In keeping with this, we observed that kisspeptin administration elicited a reduction in negative mood, providing human evidence of an antidepressant-like effect for kisspeptin, a finding with clear clinical implications.

The hippocampus is heavily involved in producing emotions. In our study, sexual- and bonding-themed stimuli resulted in positive increases in activity in the hippocampus (i.e., increases in mean percentage of blood-oxygen-level–dependent [BOLD] signal change) in line with previous studies (25, 46). In other words, the images were able to stimulate hippocampal activity. However, there was no significant difference in this increased activity between kisspeptin and vehicle administration. Overall, these data suggest that kisspeptin may have a greater effect on other limbic structures involved in emotional processing, such as the amygdala, cingulate, thalamus, and globus pallidus rather than the hippocampus.

It is salient to note that the effects of kisspeptin on the limbic system were confined to sexual and couple-bonding images, with no limbic effects in response to negative-, neutral-, happy-, or fearful-themed images. In addition, kisspeptin had no effect on brain activity during a battery of nonlimbic tasks (visual, auditory, motor, language, calculation). These data highlight that kisspeptin acts specifically to enhance limbic activity only to sexual and couple-bonding stimulation in our study, which is particularly pertinent given its established role as a potent reproductive hormone (1–3). It is also noteworthy that kisspeptin administration had no effect in the current study on other relevant hormones that could affect limbic activity, including testosterone, oxytocin, and cortisol as well as attention and anxiety. Furthermore, previous studies demonstrate that kisspeptin administration has no effect on other endocrine hormones, including growth hormone, prolactin, and thyroid-stimulating hormone in humans (47).

It is important to consider the physiological implications of our findings using the experimental paradigm employed in this study. Physiologically, kisspeptin is predominantly synthesized and secreted from kisspeptin neurones in the infundibular nucleus of the hypothalamus in humans (48) and the arcuate nucleus (ARC) and anteroventral periventricular nucleus (AVPV) in rodents (49). This kisspeptin then activates kisspeptin receptors on GnRH neurones, stimulating pulsatile GnRH release into the hypophyseal-portal circulation and downstream reproductive hormones. This secretion does appear to be pulsatile in rodents (49), and work in monkeys demonstrates pulsatile kisspeptin secretion (every 30 to 90 minutes) into the hypophyseal-pituitary circulation (50). In this study, kisspeptin was administered peripherally, as it is obviously not possible to administer it into the hypothalamus in humans, and we acknowledge that this differs from physiological kisspeptin release. However, the levels of kisspeptin achieved in this study are similar to those observed physiologically in normal pregnancy (51, 52). In addition, the kisspeptin levels observed in this study were similar to the kisspeptin levels achieved in previous studies in which peripheral kisspeptin administration stimulated oocyte maturation in in vitro fertilization protocols (53) and restored luteinizing hormone (LH) pulsatility in women with hypothalamic amenorrhoea (54). Furthermore, our study and others have demonstrated that peripheral kisspeptin administration does not result in downregulation of the reproductive axis in the time frame used in this study in healthy men (54–57). As such, while our protocol does not precisely mimic normal physiology, in the current study, we administered doses of kisspeptin that have previously been shown to have physiological and sustained reproductive effects.

Another important point to consider is whether peripherally administered kisspeptin can get into the brain. Peripheral kisspeptin can access GnRH neurones via their dendritic terminals in the organum vasculosum of the lamina terminalis (OVLT) outside the blood-brain barrier (58, 59). To examine other brain areas, we administered radiolabeled kisspeptin peripherally to male mice and demonstrated that it can access the brain, including limbic structures (Supplemental Figure 6). Although this study was performed in mice, it suggests that peripheral kisspeptin can cross the blood-brain barrier and directly access brain regions expressing kisspeptin and kisspeptin receptors (4–10). Future work will no doubt examine the neuronal pathways involved, expanding on established interactions among kisspeptin (6), GABA (60), and nitric oxide (61) pathways.

In conclusion, we implicate kisspeptin as a modulator of reproductive hormones, limbic brain activity, and behavior. This is supported by the findings of kisspeptin and kisspeptin receptor expression in limbic and paralimbic structures (4–10). We demonstrate that kisspeptin administration enhances limbic responses to sexual and bonding stimuli and that this activity correlates with reward measures, improved positive mood, and reduced sexual aversion. In addition, kisspeptin attenuates negative mood. This suggests that kisspeptin, in addition to its established role in the reproductive hormonal cascade, can also influence related sexual and emotional brain processing, thereby providing integration among reproduction, sexual responses, and bonding. These findings have important ramifications for our understanding of reproductive biology. Delineation of the precise neuronal networks by which kisspeptin exerts these effects will be an exciting field of future study, and recent advances in the use of optogenetics to stimulate endogenous kisspeptin neurones may serve as useful tools (49). Furthermore, in rodents, the kisspeptin receptor is necessary for male olfactory partner preference (62), with limbic kisspeptin neurones integrating into olfactory and reproductive circuits (6). This suggests that in humans, kisspeptin-olfactory processing may provide another area of future study.

We observed that kisspeptin’s enhancement of brain activity correlated with improvements in positive mood and reduced sexual aversion while kisspeptin also reduced negative mood. Therefore, this raises interesting directions for the pharmacological use of kisspeptin in disorders of sexual and emotional processing. For example, studies of kisspeptin administration in patients with depression and psychosexual disorders may prove fruitful as well as informing current work to develop kisspeptin as a potential therapeutic for common reproductive disorders, including male hypogonadism (56), hypothalamic amenorrhoea (54), and hyperprolactinaemia (63), and as a trigger for ovulation in in vitro fertilization (53). Therefore, our data also have important clinical relevance given the continued development of kisspeptin as a potential therapeutic.

## Methods

### Participants

Thirty-one healthy young men were recruited from advertisements in the local press following a medical screening appointment. Two participants were excluded due to excessive head motion during fMRI scanning (a priori, >2 mm), leaving a final study group of 29 healthy young men (age 25.0 ± 0.9 years). This sample size was chosen in order to give sufficient power to detect a difference in fMRI activity following a hormonal interventi on compared with vehicle. This number compares favorably with previous fMRI studies (64) and is also in line with empirically derived estimates of optimal sample sizes in fMRI studies, which suggest that an *n* of 20 to 24 is the minimal number that should give sufficient power to detect moderate-sized effects (65), and our previous work (66). In this way, we ensured adequate power to detect significant differences while also allowing for natural variation in responses.

Twenty-five participants were right-handed and 4 participants were left-handed, approximating the prevalence of left-handedness in the general population (67). While handedness can have strong effects on brain lateralization for some cognitive functions (e.g., language, spatial attention), there is no evidence that it reverses lateralization of sexual and emotional processing (68). The inclusion of both left- and right-handed participants is in line with recent recommendations to include both in neuroscience studies in order to better reflect the general population (67).

All participants were heterosexual with normal basal reproductive hormone levels (for participant characteristics, see Supplemental Table 1). Participants were free of current and past physical or psychiatric illness and were naive to psychoactive substances, prescribed or illicit, for a minimum of 6 months prior to their screening appointment. In addition, participants were excluded if there was any history of sexual aggression/abuse/phobia or psychotherapy/counselling. All participants had normal or corrected-to-normal vision.

### Study design

The 29 participants participated in 2 study visits each, as part of a randomized, double-blinded, 2-way crossover, placebo-controlled protocol (summarized in Figure 1A). This allowed participants to act as their own controls to minimize interparticipant variations in healthy physiology. All studies commenced in the morning to ensure peak basal reproductive hormone levels. Participants consumed a normal breakfast on their study days. Participants were required to abstain from alcohol, caffeine, and tobacco from midnight before their study visits. In addition, participants were asked to abstain from sexual activity from midnight before their study visits, as sexual activity prior to the study could result in changes in testosterone levels (69, 70) and residual limbic brain activity (21) as well as a postejaculatory refractory period (71) and sexual exhaustion (72).

On arrival, participants were asked to change into loose hospital scrubs and lie supine for 30 minutes to relax. Intravenous cannulae were then inserted into each antecubital fossa to allow blood collection (at time-points –30, –15, 0, 15, 30, 45, 60, and 75 minutes) and infusion of kisspeptin or vehicle. Participants completed psychometric questionnaires as detailed below. At time-point 0 minutes, a 75-minute infusion of either kisspeptin or vehicle was commenced. Participants and fMRI data analysts (L. Demetriou and M.B. Wall) were blinded as to the identity of each infusion, and the order of infusions was randomized by an independent investigator (using Research Randomizer, www.randomizer.org). Based on our previous experience of kisspeptin infusions, a dose of 1 nmol/kg/h of kisspeptin-54 was selected so as to provide steady-state levels of circulating kisspeptin from 30 to 75 minutes (during fMRI scanning and questionnaires), but avoid any increase in testosterone in this initial time frame as previously demonstrated (11, 12). Kisspeptin-54 (Bachem) was made up in gelofusine (B. Braun) and infused as previously described (12). Vehicle (gelofusine) was administered at a rate equivalent to the kisspeptin infusion.

### Assays

Blood was collected to measure circulating kisspeptin, LH, and testosterone levels, as previously described (12), and to ensure that baseline reproductive hormone levels were equivalent between study visits (Supplemental Table 2). Cortisol was measured on serum samples using an automated delayed 1-step immunoassay (Abbott Diagnostics) that uses chemiluminescent microparticle immunoassay technology. The precision of the assay was 10% or less total coefficient of variation (CV) for serum samples, with values between 83 nmol/l and 966 nmol/l. The functional sensitivity of the assay was 28 nmol/l or less, and the limit of detection was 22 nmol/l or less. Oxytocin was measured using nano–liquid chromatography–mass spectrometry (nLC-MS). The method was based on that described in Brandtzaeg et al. (73). This featured a reduction/alkylation step to liberate strongly protein-binding oxytocin, selected reaction monitoring, and a labeled internal standard, but with some modifications; for high-speed analysis (oxytocin retention time, 1.6 minutes), a short 5-mm column (200 μm ID, PepSwift, P/N 164558, Thermo Scientific) was used for both trapping and chromatographic separation. Steps were taken to ensure robust high-speed analysis. The monolithic polystyrene/divinylbenzene column material allowed for well-resolved elution of proteins still present after sample preparation. An off-line solid-phase extraction step (using Millipore C18 ZipTips; lot: R3PA16379, and elution with 30/70 ACN/0.1 % formic acid [aqueous], v/v) removed lipids prior to injection, which could have otherwise been retained by the LC stationary phase. Silica capillaries were silanized to avoid secondary interactions with the biosamples.

### Psychometric questionnaires

Participants were asked to complete a number of psychometric questionnaires. Before commencing their first study, participants completed Patient Health Questionnaire-9 to screen for depression (PHQ-9, which excluded depressive illness in our cohort as participant scores below threshold for depressive disorder) (74). State-Trait Anxiety Inventory (STAI Y2–Trait; ref. 75) excluded anxiety traits in our cohort as within normal range (76). The Behavioral Inhibition System Scale (BIS) assessed sensitivity to anticipation of punishment, and BAS assessed sensitivity to reward, desired goals, and fun (34). Greater BIS scores reflect a greater predisposition to anxiety, while greater BAS scores reflect a greater predisposition to engage in goal-directed efforts and positive feelings. The BAS scale is subdivided into 3 associated components as follows: drive, pursuit of desired goals; fun-seeking, desire for new rewards; reward, positive responses to occurrence or anticipation of reward (34). We used the following questionnaires: Sexual Desire Inventory-2 (SDI-2) to formally assess dyadic (i.e., with partner) and solitary sexual desire, which confirmed that all participants had appropriate sexual desires (77); Passionate Love Scale (PLS) to assess frequency and persistence of passionate feelings, with all participants within the average-passionate categories (78); and Love Attitudes Scale to assess individual love style (see Supplemental Table 1 for styles) (79). Participants completed a second set of questionnaires before (to confirm no baseline differences between visits, Supplemental Table 2) and during their infusions (kisspeptin or vehicle) comprising the following: Positive and Negative Affect Schedule (PANAS) involved participants scoring 20 different emotions and feelings. Higher positive-affect scores reflect greater enthusiasm, alertness, energy, and pleasurable engagement, and higher negative-affect scores reflect greater distress and unpleasurable feelings (Figure 4, B and C) (80). We also used the State-Trait Anxiety Inventory (STAI Y1-State) (75), designed to assess for any effects of the infusions on anxiety at that moment (rather than in general as in STAI Y2–Trait, as above) (Supplemental Figure 2F) (22, 75). Sexual arousal and desire were assessed during kisspeptin and vehicle infusion using the multidimensional SADI, with no differences observed (Supplemental Figure 2, A–D) (22, 81). The SADI questionnaire contains a 55-descriptor scale examining evaluative (e.g., passionate, sexy), negative (e.g. frigid, aversion), physiological (e.g., tingly, throbs in genital area), and motivational (e.g., lustful, urge to satisfy) components (81). These well-established questionnaires were selected as they have also previously been used to examine various psychometric parameters associated with reproduction that may correlate with brain activity (Supplemental Table 1) (22, 75). Finally, participants completed the D2 Test of Attention during their infusions to confirm no differences in participant concentration and attention between infusions that could have confounded activations (Supplemental Figure 2E) (75).

### MRI procedure

The MRI session consisted of the following scans: localizers, a high-resolution T1-weighted anatomical image, a B0 field-map image, the emotional images task, a resting-state fMRI scan, the emotional faces task, and finally, the fMRI battery task. The entire session lasted approximately 45 minutes. Participants used a mirror mounted on the head coil to view a screen mounted in the rear of the scanner bore, where visual stimuli were back projected through a wave guide in the rear wall of the scanner room. Participants also wore headphones in order to receive auditory stimuli and instructions from the researchers, as required. Physiological monitoring devices (pulse-oximeter and a respiratory belt) were attached to the participant, and these data were recorded using a standard data-recording system (AD Instruments PowerLab) in the control room. Infusions were performed using a Medrad Spectris Solaris MRI compatible injection system, which was also controlled using a remote panel in the control room.

#### Emotional images task.

This task used an event-related design lasting 16 minutes. Different images from the following categories were presented: sexual, nonsexual couple-bonding, negative, and neutral. The neutral and negative images were taken from the International Affective Picture System (IAPS) set (82). The sexual images were taken from a standardized set of erotic stimuli, as used previously (22). Bonding images were of happy heterosexual couples and were sourced from freely available and copyright-free stock image libraries on the internet. Each image was presented on screen for 3 seconds in a single run of 100 trials (25 trials of each image type) with a jittered inter-trial interval (ITI) of 2 to 10 seconds (based on a Poisson distribution). The participants were instructed to rate the pleasantness of each image on a 5-point scale ranging from “not at all” to “very pleasant” using a 5-key response box.

#### Emotional faces images task.

This was a block-design task lasting 12 minutes. Participants were shown faces with either happy, fearful, or neutral expressions, selected from the Karolinska Directed Emotional Faces set (83). An equal number of male and female faces were selected for the task. Each face was presented on screen for 3 seconds, and 10 faces of the same expression were presented in each 30-second block. Rest blocks (also 30 seconds) were also included, and there were 6 repetitions of each block type, presented in a pseudo-random sequence (24 blocks in total). To ensure alertness of the participants throughout the task, they were asked to respond to each face by pressing one of 2 buttons (index and middle finger) on the response box to indicate whether it was a male or female face.

#### Battery task.

This was a fast event-related task lasting 5 minutes. The experiment was adapted from Pinel et al.’s original design (84) and contained a variety of stimuli to assess different sensory and cognitive functions: visual, auditory, motor, language, and calculations. The instructions/stimuli were either presented on screen (visual) or via the headphones (auditory). The 4 trial types were as follows: (a) flashing checkboards (horizontal or vertical orientations, 20 trials); (b) simple mental calculations (audio or visual instructions, 20 trials); (c) pressing the left or right response key 3 times (visual or audio instructions, 20 trials); and (d) listening to or reading short sentences (20 trials). The combination of these 4 tasks and the variation in auditory or visual instructions allowed the mapping of 5 basic functional brain networks: visual, auditory, calculation, motor, and language (Supplemental Figure 5). The trials were presented in pseudo-randomized order in a single run of 100 trials of 3 seconds each. Randomly intermixed within the stimulus sequence were 20 null (blank screen) trials (also 3 seconds) in order to provide a baseline condition.

#### MRI acquisition.

All scanning was performed on a 3T Siemens Trio scanner with a 32-channel phased-array head coil. Anatomical images were acquired at the beginning of each scan using a T1-weighted MPRAGE pulse sequence (1 mm isotropic voxels, TR = 2300 ms, TE = 2.98 ms, flip angle = 9°). Functional images were acquired using a 3D Echo Planar Imaging (EPI) Sequence with the following parameters: TR = 2000 ms, TE1 = 13 ms, TE2 = 31 ms, flip angle = 80°, 36 axial slices, voxel size = 3 mm isotropic. The number of volumes acquired for each task differed depending on the task length: 485 for the emotional images task, 365 for the faces task, and 155 for the fMRI battery task (each task included an additional 5 volumes beyond the end of the stimulus sequence as an end-buffer period).

#### fMRI data analysis.

Image processing was performed using FSL (www.fmrib.ox.ac.uk/fsl/) version 5.0.4 (FMRIB’s software Library; Oxford Centre for Functional Resonance Imaging of the Brain [FMRIB]). Anatomical images were skull-stripped using the BET extraction tool in FSL. Only the TE = 31 images from the dual-echo sequence were analyzed, as these provide the best BOLD contrast in the majority of the brain. Functional image series were preprocessed using the following parameters: high-pass filter, 100 s, head motion correction, 6 mm (full width at half maximum [FWHM], Gaussian) spatial smoothing. Finally, the results of the analysis were coregistered to the T1 structural image of the individual and a standard anatomical template in the MNI152 space. For the analysis of all the tasks, a standard general linear model (GLM) was used, as implemented in the FEAT module in FSL. Regressors derived from the onset times of each stimulus condition were convolved with a gamma function in order to simulate the hemodynamic response function (HRF). Regressors derived from head-motion parameters were also included in the first-level models as regressors of no interest. For the event-related tasks (emotional images and fMRI battery tasks), the first temporal derivatives of each stimuli time series were also included. Contrasts were defined that isolated activity related to each stimulus condition relative to the baseline and also compared between 2 stimulus conditions, as appropriate. Two sets of group level models computed the mean task/stimuli-related activation across all participants and all scans and compared the 2 treatment conditions. A regressor of no interest was included in the latter analyses to model the treatment/session order in order to control for any potential order effects. A mixed-effects (FLAME-1) model was used to enable generalization of results to the population. A statistical threshold of *Z* = 2.3 (*P* < 0.05 cluster-corrected for multiple comparisons) was used for all group analyses.

A priori anatomical limbic and paralimbic ROIs were selected for further analysis. These were the amygdala, hippocampus, anterior and posterior cingulate, thalamus, globus pallidus, and putamen based on the expression pattern of *KISS1/KISS1R* in the limbic and paralimbic system in humans (9, 10) and established structures involved in sexual and emotional processing (15, 17–19, 21–28, 38, 39, 43, 46, 85–87) (Supplemental Figure 1). ROIs were defined in standard stereotactic space using the Harvard-Oxford cortical and subcortical atlases (distributed by https://fsl.fmrib.ox.ac.uk/fsl/fslwiki/). The mean of all voxel values within each ROI was extracted from the brain images for each participant per session, and a statistical analysis was performed as below.

### Statistics

Statistical analyses were performed by a statistician (P. Bassett). Data were normally distributed by Kolmogorov testing. Hormone level data analysis was performed using 2-way ANOVA. Baseline and treatment effect psychometric data were analyzed using multilevel linear regression. Two-level models were used, with individual measurements contained within participants. Terms in the model included both treatment (kisspeptin or vehicle) and visit order. fMRI data task analysis was performed using a GLM with regressors (see details in Supplemental Methods). A statistical threshold of *Z* = 2.3 (cluster corrected for multiple comparisons) was used for all group fMRI analyses. For fMRI ROI analysis, a 2 (stimulus, bonding vs. sexual) by 2 (treatment, kisspeptin vs. vehicle) by 8 (ROI) repeated-measures ANOVA revealed significant differences for all the main effects and 2-way interactions (*P* < 0.01) except for the stimulus by treatment interaction. The 3-way interaction was also significant (*F* = 2.51, *P* < 0.05). Post-hoc paired 2-sided *t* tests were conducted to reveal the details of these interactions. Correlations were assessed using partial correlation with associations adjusted for visit order. An α threshold of *P* < 0.05 identified statistical significance except for the correlation analyses, where the threshold was reduced to *P* < 0.01 (to adjust for the number of analyses performed).

### Study approval

The human study was performed in accordance with the Declaration of Helsinki. All participants gave written informed consent prior to inclusion in the study. The study was approved by the regional ethics committee (National Research Ethics Service London, United Kingdom, REC ref: 04/Q0406/151). The animal study was approved by the King’s College London Animal Welfare and Ethical Review body, and experiments were carried out in accordance with the Animal Scientific Procedures Act (1986) and Amendment Regulations 2012.

## Author contributions

ANC, MBW, CNJ, EAR, SRB, and WSD conceived the study. ANC, MBW, LD, AJS, SAC, SN, AN, CIE, JKP, AA, RR, VS, GMN, MT, RCB, and SAT collected the data. ANC, MBW, LD, AJS, PB, AM, OKB, EL, SRW, MRS, CH, RCB, and SAT analyzed the data. ANC, MBW, LD, and WSD wrote the manuscript.

## Supplementary Material

Supplemental data

ICMJE disclosure forms
