# Supplementary material for: Kisspeptin modulates sexual and emotional brain processing in humans
Source: J Clin Invest. 2017 Jan 23;127(2):709–19. doi: 10.1172/JCI89519 (PMC5272173; doi:10.1172/JCI89519)
Supplement: Supplemental data [file jci-127-89519-s001.pdf]

Supplemental Data: Figures, Tables, Methods and References.

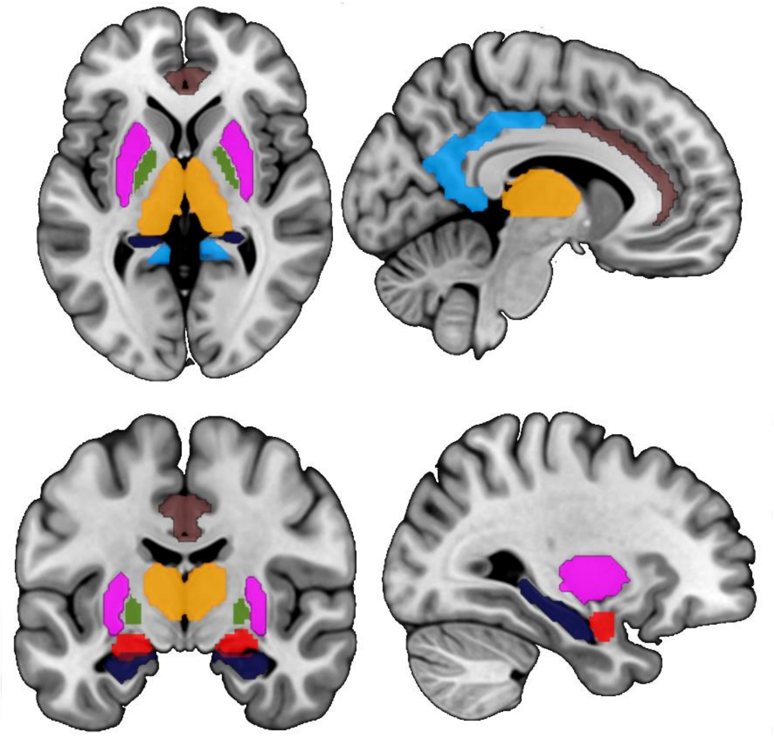

Supplemental Figure 1. A priori anatomically-defined region of interest (ROI) masks. Amygdala (red), hippocampus (dark blue), thalamus (yellow), anterior cingulate (brown), posterior cingulate (light blue), globus pallidus (green), putamen (pink). All ROIs were derived from the Harvard-Oxford cortical and sub-cortical atlases, thresholded at 0.25 and shown here overlaid on the MNI152 stereotactic template brain.

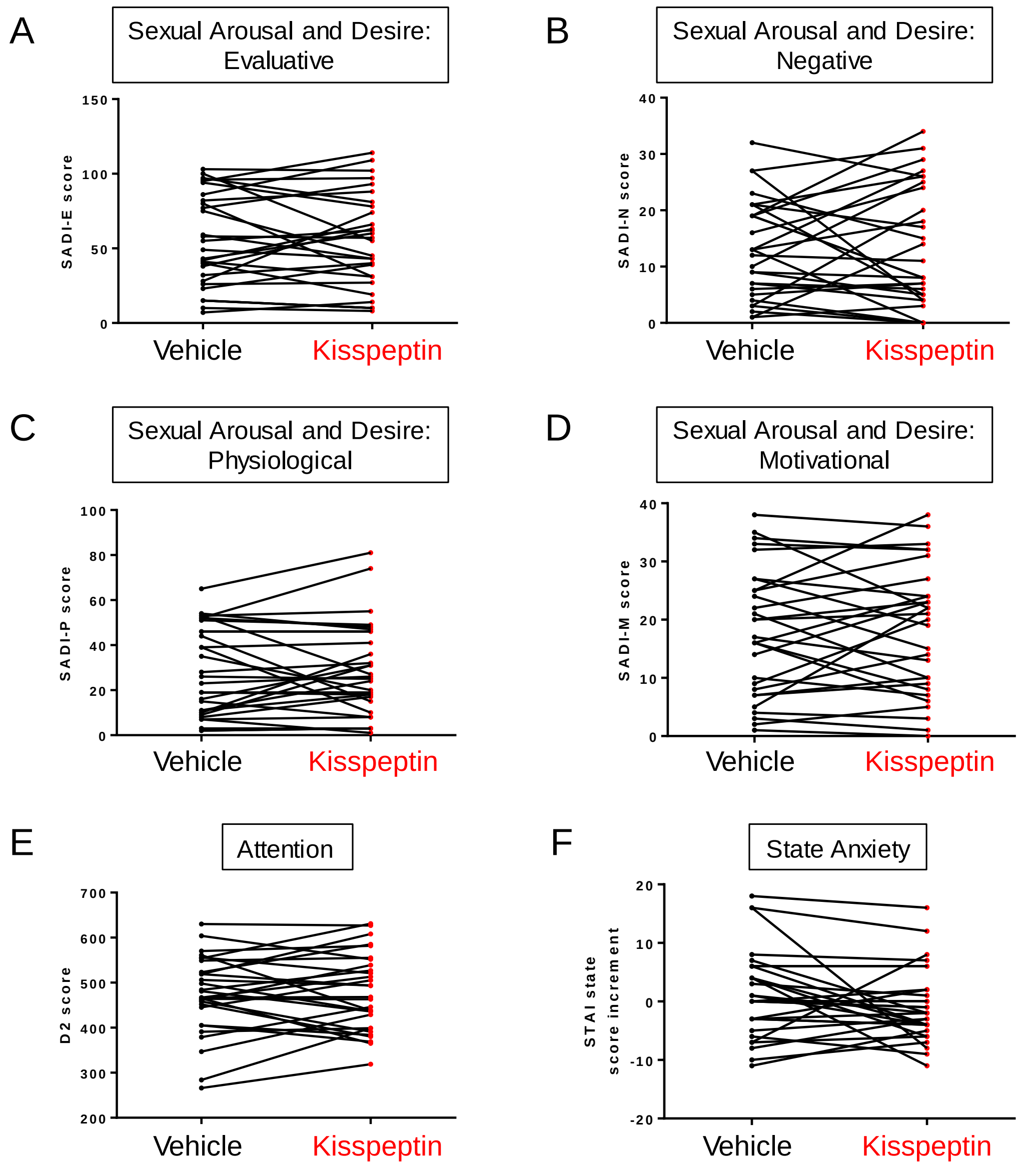

Supplemental Figure 2. Effects of kisspeptin administration on sexual arousal and desire, attention and anxiety. Sexual arousal and desire were assessed during infusion of kisspeptin and vehicle using the Sexual Arousal Desire Inventory (SADI). Kisspeptin infusion did not alter (**A**) evaluative; (**B**) negative; (**C**) physiological; (**D**) motivational descriptors. (**E**) Attention was assessed using the D2 test of attention during the infusion and was unaltered by kisspeptin infusion compared to vehicle. (**F**) State anxiety before and during the infusion was assessed using the State-Trait Anxiety Inventory (State component, Form Y) and was unaltered by kisspeptin infusion compared to vehicle. Multi-level linear regression, adjusted for visit order. *n* = 29.

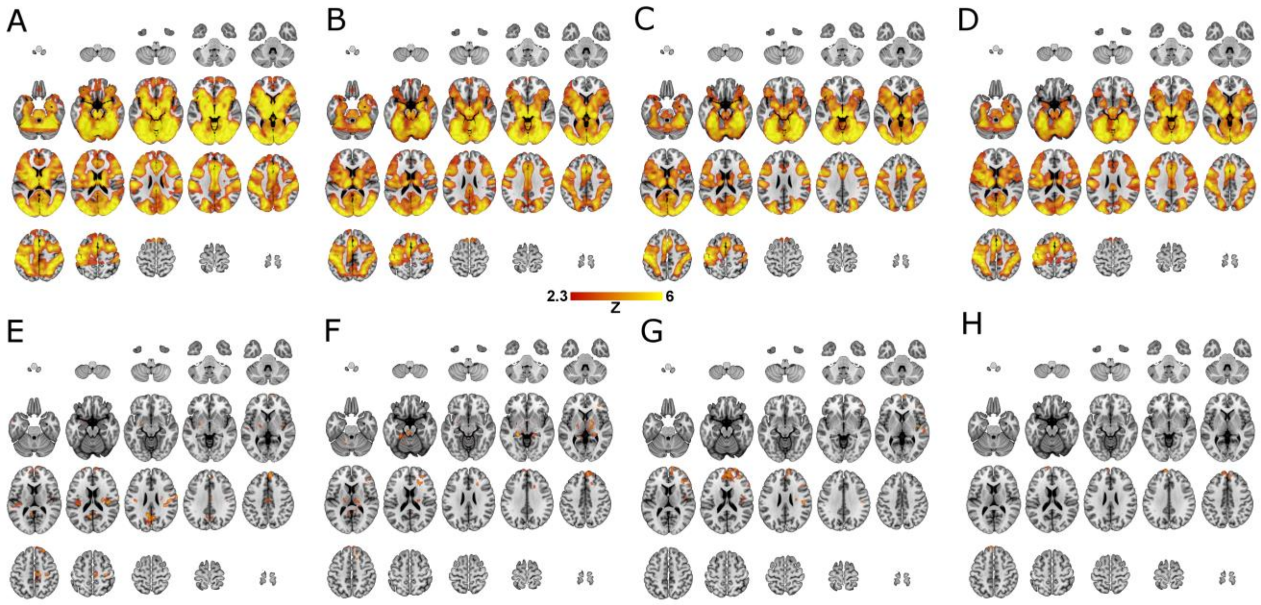

Supplemental Figure 3. Task effects and kisspeptin modulation of these effects in response to emotional-themed images (emotional images task). (**A-D**) Analyses of the main effects of stimulus type (all subjects, both treatments averaged) for sexual, non-sexual couple-bonding, negative, and neutral images (A-D, respectively) relative to baseline. (**E-H**) Within-subject analyses of the effect of kisspeptin vs. vehicle for sexual, non-sexual couple-bonding, negative, and neutral images (E-H, respectively). Positive voxel values represent an increase in activity in the kisspeptin treatment. All statistical maps thresholded at $Z = 2.3$, $P < 0.05$ (cluster corrected for multiple comparisons), $n = 29$.

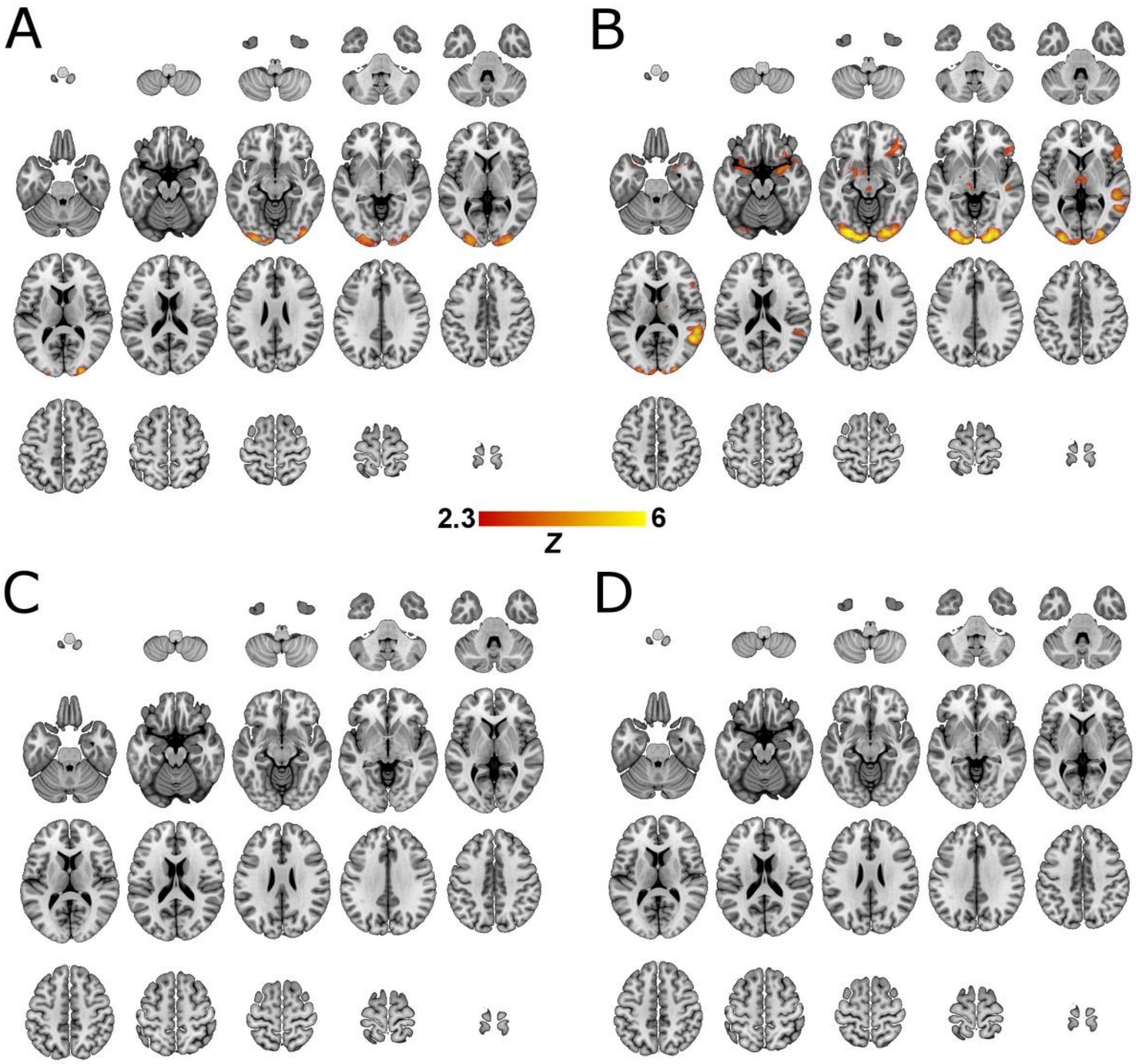

Supplemental Figure 4. Task effects and kisspeptin modulation of these effects in response to happy and fearful faces (emotional faces images task). Analyses of the main effects of stimulus type (all subjects, both treatments averaged) for happy (**A**) and fear (**B**) facial expressions, both relative to a neutral expression control condition. Within-subjects analyses of the effect of kisspeptin vs. vehicle on happy (**C**) and fear (**D**) facial expressions, relative to neutral expression control. No significant voxels are evident in the treatment analysis. All statistical maps thresholded at $Z = 2.3$, $P < 0.05$ (cluster corrected for multiple comparisons), $n = 29$.

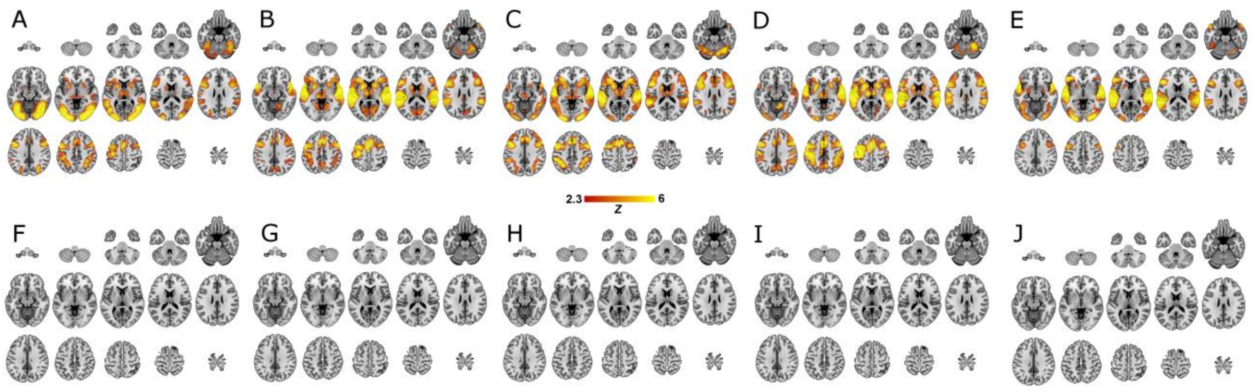

Supplemental Figure 5. Task effects and kisspeptin modulation of these effects in response to a battery of non-limbic tasks. (**A-E**) Analyses of the main effects of stimulus type (all subjects, both treatments averaged) for the fMRI battery control task, with visual, auditory, calculation, motor, and language components (A-E, respectively). (**F-J**) Within-subject analyses of the effect of kisspeptin vs. vehicle on visual, auditory, calculation, motor, and language components of the task (F-J, respectively). No significant voxels are evident in the treatment analysis, on any of the components of the task. All statistical maps thresholded at $Z = 2.3$, $P < 0.05$ (cluster corrected for multiple comparisons), $n = 29$.

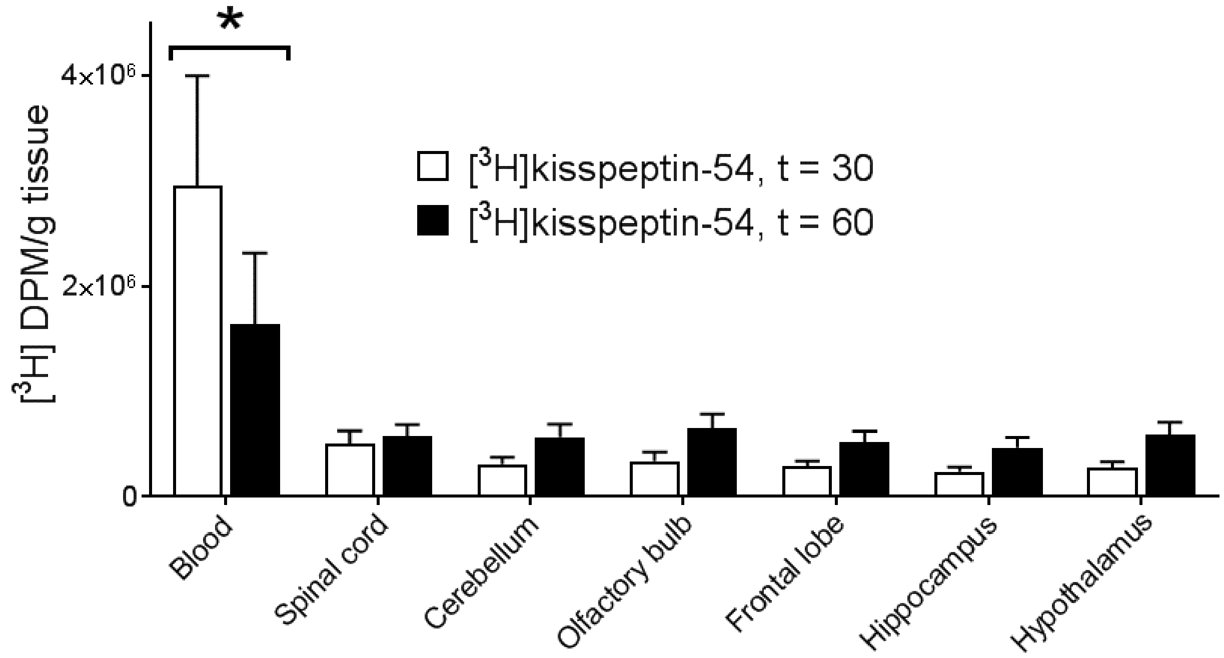

Supplemental Figure 6. Peripherally administered radiolabelled kisspeptin-54 can cross into the adult male mouse brain. The ability of peripherally (i.p.) administered [^{3}H]kisspeptin-54 to get into the brain was assessed by regional dissection analysis as described in the Supplemental Methods below. Samples of spinal cord, cerebellum, olfactory bulb, frontal lobe, hippocampus and hypothalamus were weighed and solubilised, and [^{3}H]kisspeptin-54 levels were determined by liquid scintillation counting. Results demonstrate that [^{3}H]kisspeptin-54 from the blood can get into the brain, as evidenced by the presence of radioactivity in the regions investigated. Furthermore, whilst the level of [^{3}H]kisspeptin-54 in the blood decreased significantly from 30 to 60 min, concentrations in the brain structures were maintained during this period, indicative of accumulation of [^{3}H]kisspeptin-54 in the brain. Data depicts mean ± s.e.m., one-way ANOVA with post-hoc Bonferonni correction, time only significantly changed blood concentration, * $P < 0.05$, C57BL/6 adult male mice, n = 4-7.

Supplemental Table 1: Participant clinical and psychometric characteristics.

		Healthy Men (n = 29)
		Mean ± SEM
Age (years)		25.0 ± 0.9
BMI (kg/m^2)		23.6 ± 0.4
Baseline Reproductive Hormones		
	Kisspeptin (pmol/L)	33.7 ± 6.7
	LH (IU/L)	3.6 ± 0.3
	FSH (IU/L)	2.8 ± 0.2
	Testosterone (nmol/L)	21.3 ± 1.2
Number of sexual partners in last year		2.3 ± 0.4
Frequency of sexual intercourse per month		7.3 ± 1.5
Hours viewing pornographic material per week		0.8 ± 0.1
PHQ-9 (Depression Screen)		2.0 ± 0.4
PANAS		
	Positive Affect	33.4 ± 1.3
	Negative Affect	12.5 ± 0.4
STAI-Y Trait		33.1 ± 1.2
BIS		18.4 ± 0.7
BAS		
	Drive	11.2 ± 0.4
	Fun	11.7 ± 0.4
	Reward	17.2 ± 0.3
PLS		94.8 ± 4.7
SDI-2		
	Dyadic	48.3 ± 2.0
	Solitary	16.0 ± 1.2
	Total	68.2 ± 3.2
Love Attitudes Scale		
	Eros (passionate love)	1.8 ± 0.1
	Ludus= (game-playing love)	3.4 ± 0.2
	Storge (friendship love)	2.8 ± 0.3
	Pragma (practical love)	3.7 ± 0.2
	Mania (possessive love)	3.4 ± 0.2
	Agape (altruistic love)	2.5 ± 0.2

BMI = Body Mass Index; LH = Luteinising Hormone; FSH = Follicle Stimulating Hormone; PH-9 = Patient Health Questionnaire-9 to screen for depression (not observed in any participant) (1); PANAS = Positive And Negative Affect Schedule for assessment of mood (2); STAI-Y Trait = State-Trait Anxiety Inventory (Form Y) to assess trait anxiety (3); BIS = Behavioural Inhibition System Scale to assess sensitivity to anticipation of punishment; BAS = Behavioural Activation System Scale to assess sensitivity to desired goals, fun and reward (4); PLS = Passionate Love Scale to assess frequency and persistence of passionate feelings (5); SDI = Sexual Desire Inventory to assess dyadic (i.e. with partner) and solitary sexual desire (6). Love Attitudes Scales reflects individual love style (7). Screening ensured no active depression or anxiety trait that could affect fMRI analysis. Data presented as mean ± s.e.m.

Supplemental Table 2: Participant baseline clinical and psychometric characteristics at kisspeptin and vehicle visits.

		Kisspeptin Visit (n = 29)	Vehicle Visit (n = 29)
		Mean ± SEM	Mean ± SEM
Baseline Reproductive Hormones			
	Kisspeptin (pmol/L)	28.0 ± 6.9	33.9 ± 3.9
	LH (IU/L)	3.7 ± 0.3	3.6 ± 0.3
	FSH (IU/L)	2.7 ± 0.2	2.7 ± 0.2
	Testosterone (nmol/L)	21.0 ± 1.3	19.5 ± 1.1
PANAS			
	Positive Affect	33.6 ± 1.4	33.4 ± 1.4
	Negative Affect	13.2 ± 0.6	12.4 ± 0.5
STAI-Y State		30.8 ± 1.2	30.5 ± 1.3
PLS		96.6 ± 4.7	98.2 ± 4.4

LH = Luteinising Hormone, FSH = Follicle Stimulating Hormone, PANAS = Positive And Negative Affect Schedule, STAI-Y State = State-Trait Anxiety Inventory (Form Y), PLS = Passionate Love Scale. 15 participants received kisspeptin and 16 received vehicle at first visit. No significant difference in parameters between visits assessed by multi-level linear regression, adjusted for visit order. Data presented as mean ± s.e.m..

Supplemental Table 3: Structures with enhanced activation by kisspeptin on whole brain analysis.

Limbic and Basal Ganglia Brain Structures:

	Image Theme			
Structure	Sexual	Couple-Bonding	Negative	Neutral
Amygdala	-26,-2,-16	-16,-2,-20		
Thalamus	-18,-20,8	-12,-32,-2; 16,-32,-2		
Posterior Cingulate Cortex	-8,-56,28; 8,-60,26	-6,-52,8; 8,-54,16		
Mid-Cingulate Gyrus	4,-2,36; -4,-18,40			
Hippocampus		-24,-36,-2; 22,-36,-2		
Putamen	-28,-6,-8; 32,-12,2	-28,-4,-6; 32,-10,2		
Globus Pallidus	-22,-10,4	-22,-10,2; 20,-6,2		

Other Structures:

	Image Theme			
Structure	Sexual	Couple-Bonding	Negative	Neutral
Precentral Gyrus	34,-22,54			
White Matter	26,-14,22	24,26,24		
Frontal Pole	22,40,54			-10,52,46
Superior Frontal Gyrus	6,46,36	4,48,42	-2,54,18; 2,54,18	4,46,42
Medial Frontal Gyrus			46,24,24	
Supramarginal Gyrus			50,-22,28	
Heschl's Gyrus	-54,-16,4			
Heschli's Gyrus			44,12,6	

Data derived from whole brain analysis during emotional images task on viewing sexual, non-sexual couple-bonding, negative and neutral themed images (n = 29). Co-ordinates represented by x, y, z (left and right alignment within a column represents left and right hemispheres respectively) and are derived from the MNI152 stereotactic coordinate space. Coordinates represent the centre of discrete activation clusters observed in the group-level analyses of treatment effects (kisspeptin vs. vehicle), with statistical maps thresholded at Z = 2.3, $P < 0.05$ (cluster-corrected for multiple comparisons).

Supplemental Methods

[^{3}H] Kisspeptin-54 animal method

Male C57BL/6 mice (24.7 ± 0.47 g, 8-10 weeks) were obtained from Harlan UK Ltd (Bicester, Oxon, UK) and allowed to acclimatise to the Biological Services Unit at King's College London for at least 3 days before treatment. Kisspeptin-54 trifluoroacetate salt (molecular weight 5857.51 g/mol; purity >95%) was purchased from Bachem and was custom tritiated by Quotient Bioresearch (Radiochemicals) Ltd. and stored in ethanol (specific activity: 88 Ci/mmol, radiochemical purity 99.7%). Mice were injected i.p. with [^{3}H-propionylated]kisspeptin-54 (0.04 nmol/g animal weight) dissolved in physiological saline. After 30 or 60 minutes, the mice were sacrificed by anaesthetic overdose (pentobarbitone), heparinised (100 U i.p.) blood samples were taken via cardiac puncture, and the brains were removed. Regional dissection was performed to isolate samples of the spinal cord, cerebellum, olfactory bulb, frontal lobe, hippocampus, and hypothalamus. These structures were selected to ensure accurate anatomical dissection.

All tissue samples were weighed before processing for liquid scintillation counting. Samples were solubilised over 48-72 hours in 0.5 ml Solvable (PerkinElmer Life Science) at 37 C. After vortexing, 4 mls of scintillation fluid was added to each sample, and radioactivity for [^{3}H] was estimated using a Packard TriCarb 2900TR counter according to previously published methods (1-3). All results were corrected for background radioactivity, and expressed as mean ± s.e.m. (DPM/g tissue sample, n = 4-7).

In order to determine the free [^{3}H] label, we precipitated samples of stock solution and plasma with 100% trichloroacetic acid (v:v 4:1, sample:TCA). After spinning the precipitates at 13,000 rpm for 15 min at 4°C, levels of radioactivity in the supernatant were assessed by liquid scintillation counting and compared to the total radioactivity in a parallel sample that had not been precipitated with TCA. The presence of free label [^{3}H] in the plasma ranged from 23-55% at 30 minutes and 29-90% at 60 minutes (n = 4).

Supplemental References

1 Mason BL, Pariante CM, Thomas SA. A revised role for P-glycoprotein in the brain distribution of dexamethasone, cortisol, and corticosterone in wild-type and ABCB1A/B-deficient mice. *Endocrinology.* 2008;149(10):5244-53.

2 Sanderson L, Dogruel M, Rodgers J, De Koning HP, Thomas SA. Pentamidine movement across the murine blood-brain and blood-cerebrospinal fluid barriers: effect of trypanosome infection, combination therapy, P-glycoprotein, and multidrug resistance-associated protein. *J Pharmacol Exp Ther.* 2009;329(3):967-77.

3 Jeganathan S, Sanderson L, Dogruel M, Rodgers J, Croft S, Thomas SA. The distribution of nifurtimox across the healthy and trypanosome-infected murine blood-brain and blood-cerebrospinal fluid barriers. *J Pharmacol Exp Ther.* 2011;336(2):506-15.
